# Directionally non-rotating electric field therapy delivered through implanted electrodes as a glioblastoma treatment platform: A proof-of-principle study

**DOI:** 10.1093/noajnl/vdae121

**Published:** 2024-07-13

**Authors:** Jun Ma, Shilpi Singh, Ming Li, Davis Seelig, Gregory F Molnar, Eric T Wong, Sanjay Dhawan, Stefan Kim, Logan Helland, Hsien-Chung Chen, Nikos Tapinos, Sean Lawler, Gatikrushna Singh, Clark C Chen

**Affiliations:** Department of Neurosurgery, University of Minnesota, Minneapolis, Minnesota, USA; Department of Neurosurgery, University of Minnesota, Minneapolis, Minnesota, USA; Department of Neurosurgery, University of Minnesota, Minneapolis, Minnesota, USA; Department of Veterinary Clinic Sciences, College of Veterinary Medicine, University of Minnesota, St. Paul, Minnesota, USA; SynerFuse Inc., Eden Prairie, Minnesota, USA; Department of Neurology, Warren Alpert School of Medicine, Rhode Island Hospital, Brown University, Providence, Rhode Island, USA; Department of Neurosurgery, University of Minnesota, Minneapolis, Minnesota, USA; Department of Neurosurgery, University of Minnesota, Minneapolis, Minnesota, USA; Department of Neurosurgery, University of Minnesota, Minneapolis, Minnesota, USA; Department of Neurosurgery, Department of Neurosurgery, Shuang Ho Hospital, Taipei Medical University, Taipei, Taiwan; Department of Neurosurgery, Warren Alpert School of Medicine, Rhode Island Hospital, Brown University, Providence, Rhode Island, USA; Department of Pathology and Laboratory Medicine, Legorreta Cancer Center, Brown University, Providence, Rhode Island, USA; Department of Neurosurgery, University of Minnesota, Minneapolis, Minnesota, USA; Department of Neurosurgery, University of Minnesota, Minneapolis, Minnesota, USA; Department of Neurosurgery, Warren Alpert School of Medicine, Rhode Island Hospital, Brown University, Providence, Rhode Island, USA

**Keywords:** directionally non-rotating electric field therapy (dnEFT), electric stimulator electrodes, glioblastoma, macrophages, microglia

## Abstract

**Background:**

While directionally rotating tumor-treating fields (TTF) therapy has garnered considerable clinical interest in recent years, there has been comparatively less focus on directionally non-rotating electric field therapy (dnEFT).

**Methods:**

We explored dnEFT generated through customized electrodes as a glioblastoma therapy in in vitro and in vivo preclinical models. The effects of dnEFT on tumor apoptosis and microglia/macrophages in the tumor microenvironment were tested using flow-cytometric and qPCR assays.

**Results:**

In vitro, dnEFT generated using a clinical-grade spinal cord stimulator showed antineoplastic activity against independent glioblastoma cell lines. In support of the results obtained using the clinical-grade electrode, dnEFT delivered through a customized, 2-electrode array induced glioblastoma apoptosis. To characterize this effect in vivo, a custom-designed 4-electrode array was fabricated such that tumor cells can be implanted into murine cerebrum through a center channel equidistant from the electrodes. After implantation with this array and luciferase-expressing murine GL261 glioblastoma cells, mice were randomized to dnEFT or placebo. Relative to placebo-treated mice, dnEFT reduced tumor growth (measured by bioluminescence) and prolonged survival (median survival gain of 6.5 days). Analysis of brain sections following dnEFT showed a notable increase in the accumulation of peritumoral macrophage/microglia with increased expression of M1 genes (IFNγ, TNFα, and IL-6) and decreased expression of M2 genes (CD206, Arg, and IL-10) relative to placebo-treated tumors.

**Conclusions:**

Our results suggest therapeutic potential in glioblastoma for dnEFT delivered through implanted electrodes, supporting the development of a proof-of-principle clinical trial using commercially available deep brain stimulator electrodes.

## Graphical Abstract

**Graphical Abstract:**
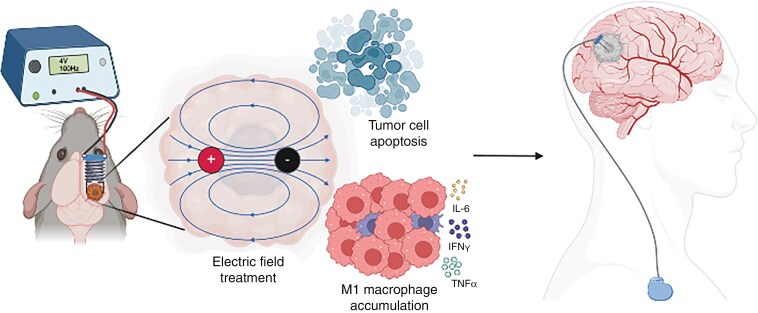
Directionally non-rotating electric field treatment (dnEFT) generated through electrodes designed for intracranial implant induces glioblastoma apoptosis and accumulation of M1 macrophages/microglia in the tumor microenvironment of preclinical models. These results support consideration for a proof-of-principle clinical trial using commercially available deep brain stimulator electrodes in the near future.

**Key Points**

- Directionally non-rotating electric field therapy (dnEFT) generated through electrodes designed for intracranial implant-induced anti-glioblastoma effects.
- Pertaining to the tumor, dnEFT caused glioblastoma apoptosis.
- Pertaining to the tumor microenvironment, dnEFT led to an accumulation of macrophage/microglia with increased expression of M1 genes.

**Importance of the Study**

The FDA-approved directionally rotating tumor-treating field (TTF) employs scalp electrodes that generate the highest field intensity at the scalp, posing challenges in accurately targeting of deep-seated tumors. This study examines an approach using directionally non-rotating electric field therapy (dnEFT) generated through electrodes designed for intracranial implantation. Our in vitro and in vivo results indicate that dnEFT induced significant anti-glioblastoma effects, including tumor apoptosis and antitumor immunity. Direct electrode implant into glioblastoma-bearing regions of the cerebrum affords precise targeting of dnEFT and associated anti-glioblastoma effects. The potential for the clinical translation of this promising approach into a proof-of-principle human study is enhanced by the ready availability of clinical-grade deep brain stimulator electrodes, currently implanted in the brain as treatment for neurodegenerative diseases.

Electrostatic interactions constitute a fundamental physical force that mediates the dynamic interplay between nucleic acids, proteins, and lipids that is essential for life.^1^ Modulation of these interactions through imposition of external electric fields (termed electric field therapy, or EFT) generated through alternating currents has long been proposed as a cancer therapeutic strategy.^2–5^ Given the multiplicity of cellular interactions modulated by electrostatic forces, it is not surprising that EFT is associated with pleiotropic effects, ranging from induction of mitotic catastrophe^6–8^ and altered protein interactions,^5,9^ to disruption of membrane dynamics.^10^ Further, high-frequency EFT (>500 kHz) causes vibration of polar molecules, leading to hyperthermic effects.^11^ There is growing interest in harnessing the antineoplastic effects of EFT as a tumor-treating strategy, especially after the U.S. Food and Drug Administration (FDA) approval of directionally rotating tumor-treating field (TTF) as a treatment for glioblastomas in 2015.^12^

Glioblastoma is the most common form of malignant primary brain cancer in adults and remains one of the deadliest human tumors.^13^ Fatality within 2 years of diagnosis is nearly universal,^14,15^ and there is currently no consensus on the optimal treatment for recurrent glioblastomas refractory to the standard-of-care concurrent temozolomide-radiation treatment. TTF was initially evaluated in patients with recurrent glioblastoma. In a phase III randomized clinical trial (RCT), the median overall survival of recurrent glioblastoma patients receiving TTF was ~6 months and comparable to those receiving chemotherapy.^9,14^ A subsequent RCT evaluated the addition of TTF to the standard-of-care therapy for patients with newly diagnosed glioblastoma. This study showed that the median survival of the group treated with standard-of-care augmented with TTF was 19.2 months, which was statistically significantly longer than the 16.2 months observed in the group receiving standard-of-care therapy only. These RCTs led to the FDA approval of TTF for recurrent and newly diagnosed glioblastoma.^16,17^

The current, FDA-approved TTF device consists of scalp electrodes producing directionally rotating electric fields, which were initially proposed to disrupt chromosomal alignment during mitosis.^6^ Subsequent studies showed TTF induced pleiotropic effects beyond mitotic perturbation.^5,18^ Of note, antineoplastic effects have also been reported when cancer cells are subjected to a directionally non-rotating electric field (dnEF).^3–5,19^ Such dnEF can be generated by surgically implanting electrodes into the cancer-bearing regions.^4,20^ A key advantage of this strategy is that the dnEF is most intense in the region of the tumor, where the electrode is inserted. In contrast, the highest field intensity for a scalp electrode is generated at the scalp^21,22^ and some patients suffer skin irritation or breakdown during TTF treatment.^19^ Moreover, it is difficult to precisely target the desired field for deep-seated tumors given (1) the anatomic space between the scalp electrode and the target lesion, (2) structures of varying histology that presides in this space, and (3) the low-frequency electric stimulation associated with TTF.^21–23^ Importantly, clinical-grade electrodes are currently intracranially implanted as treatment for neurodegenerative diseases.^24,25^ Such electrodes may be repurposed for glioblastoma treatment as proof-of-principle in a first-in-human study.

While the concept of dnEFT through surgically implanted electrodes is compelling from a theoretical perspective,^3–5,19,26,27^ there is little in the current literature to support its application as a glioblastoma therapy. In this study, we demonstrate that tumoricidal activity against glioblastoma can be achieved through dnEFT both in vitro and in vivo, providing a foundation for future clinical translation.

## Materials and Methods

### Cell Lines and Culture

Human glioblastoma cell lines LN340 (kindly gifted by Dr. Lynda Chin at University of Texas), H1915 (ATCC), and GL261 (ATCC), normal human astrocytes (NHA, ATCC) were cultured in Dulbecco’s modified Eagle’s medium (DMEM) supplemented with 10% fetal bovine serum (FBS), 2 mM l-glutamine and 1% penicillin/streptomycin in a humidified atmosphere at 37°C with 5% CO_2_. MGG 123 cells, a patient-derived glioblastoma spheroid line,^28^ were cultured in neurobasal medium (Gibco) supplemented with B27, FGF, and EGF growth factors. The identity of each cell line was verified using short tandem repeat profiling^29^ on June 4, 2023.

### In vitro Directionally Non-Rotating Electrode Field Therapy Experiments

**Figure 1.**
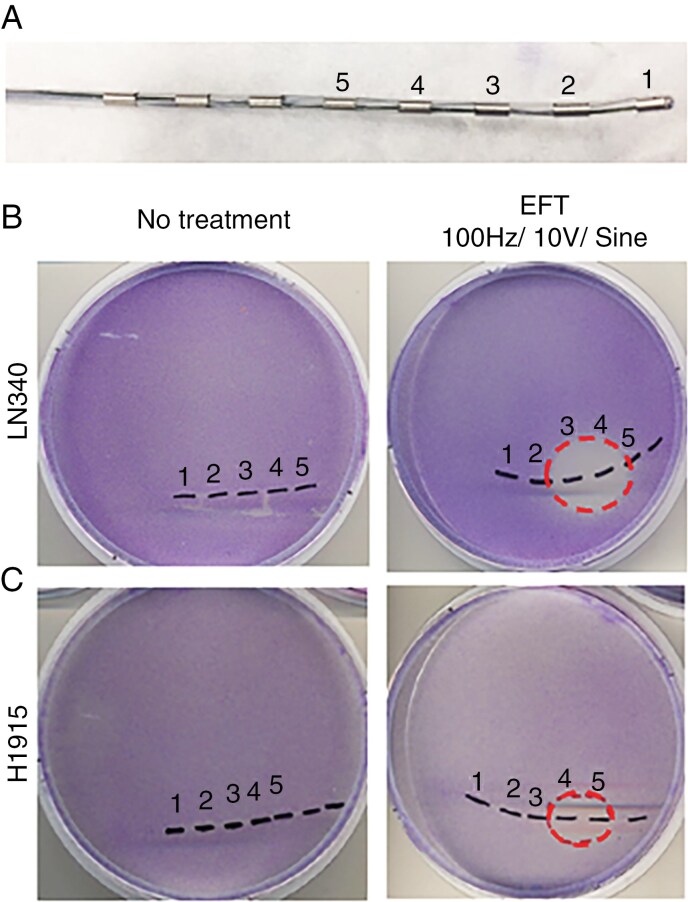
Antineoplastic effect associated with directionally non-rotating electric field (dnEF) exposure. (A) Commercial clinical-grade percutaneous spinal cord stimulation lead (Model No. 3186; 3 mm contact length/4 mm spacing, Abbott Neuromodulation) with multiple contacts (silver stripes) along the white catheter. Electrical contacts were numerically labeled 1 to 5. (B) Absence of detectable glioblastoma cells in the region exposed to dnEFT. LN340 cells were seeded onto a 10-cm tissue culture dish with affixed catheter electrode and allowed to grow to confluency while dnEFT (alternating current, 100 Hz, 4 V, sine wave) was generated between contacts 3 and 4. The locations of the contacts were marked by the dotted line and labeled 1 to 5. The region of the dnEFT induction is marked in dotted circle. (C) Absence of detectable H1915 (non–small lung cancer cell line) in the region exposed to dnEFT. H1915 cells were seeded onto a 10-cm tissue culture dish with affixed catheter electrode and allowed to grow to confluency while dnEFT (alternating current, 100 Hz, 4 V, sine wave) was generated between contacts 4 and 5. The locations of the contacts were marked by the dotted line and labeled 1 to 5. The region of the dnEFT induction (100 Hz, 4 V, sine wave) is marked in dotted circle. Of note, the fixation of the electrode was imperfect for the LN340 experiment, and there were minor movements of the electrode, leading to the lack of cells in regions previously occupied by the electrode.

**Figure 2.**
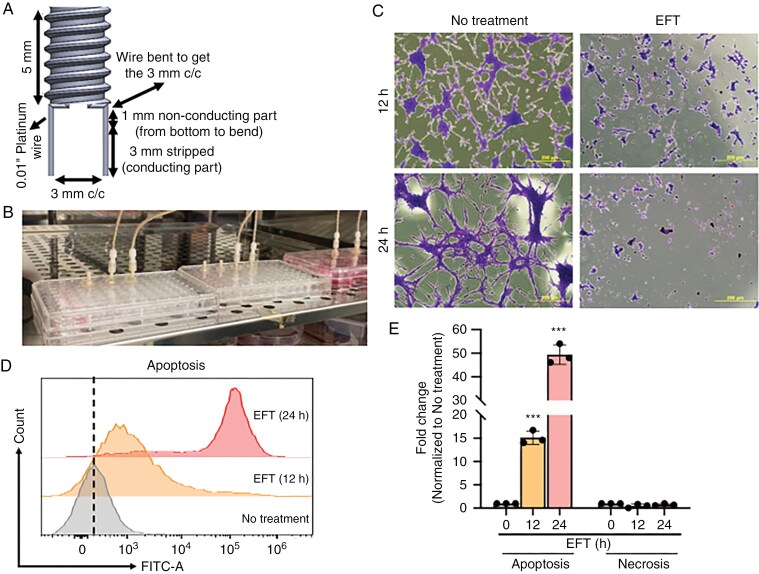
Directionally non-rotating electric field treatment (dnEFT) induces glioblastoma apoptosis in vitro. (A) Schematic of the custom-designed 2-electrode array compatible with the 96-well tissue culture plate. (B) Photo of the 2-electrode array affixed to the cover plate of the 96-well culturing dishes. (C) Crystal violet staining LN340 after 12 and 24 h of dnEFT. (D) Flow-cytometric analysis of Annexin V-FITC and propidium iodide-stained MGG123 glioblastoma primary sphere lines with or without dnEFT (100 Hz, 4 V, sine wave, 12 or 24 h). Histogram represents the Annexin V-FITC-positive apoptotic cell distribution of no-treatment, EFT treatment at 12 h and 24 h. (E) The fold change of apoptotic and necrotic cells population was determined by the normalization with unstimulated cell population. The bar graph represents the mean standard deviation of 3 independent biological replicates. Statistical significance: ***P ≤ .001.

**Figure 3.**
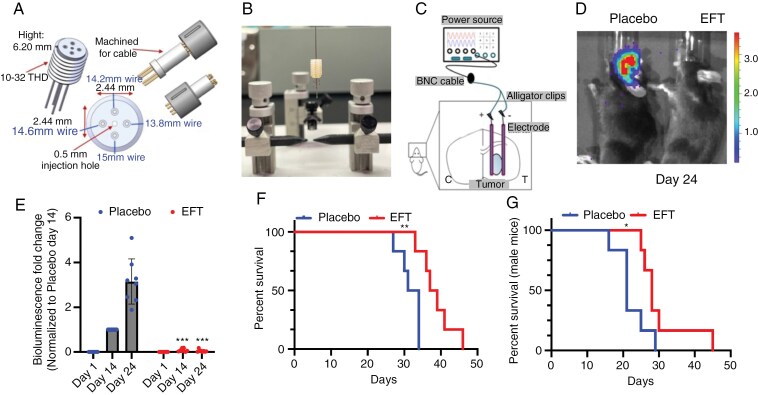
Directionally non-rotating electric field treatment (dnEFT) suppressed glioblastoma growth in vivo. (A) Schematic of the custom-designed 4-electrode array with a central channel for implant of glioblastoma cells. (B) Photo of the 4-electrode array complex prior to implant onto the murine cranium. (C) Schematic of the implanted tumors centrally located within the electric field defined by the 4-electrode array. (D) dnEFT suppressed glioblastoma growth in vivo. Bioluminescence (BLI) of mice implanted with the 4-electrode array and luciferase-labeled GL261 (Luc-GL261) with and without dnEFT. Representative BLI image is shown in (D). (E) Serial BLIs at Days 1, 14, and 24. Of note, Day 1 BLI was measured on the first day of dnEFT, which is 7 days after the tumor implant, and the value of the BLI on Day 1 is not zero—just very low relative to the Day 24 BLI. One week after Luc-GL261 implant and verification of uptake by bioluminescence, C57BL/6 female mice were randomized dnEFT (100 Hz, 4 V, sine wave) or placebo treatment for 2 weeks and scanned for bioluminescence subsequently. *n* = 8 for dnEFT and placebo-treated groups. Statistical significance: ***P ≤ .001. (F) Kaplan–Meier analysis of dnEFT and placebo treated C57BL/6 female mice (*P ≤ .01.) or (G) male mice (*P ≤ .05) treated as described (E). *n* = 6 for dnEFT and placebo-treated male mice.

**Figure 4.**
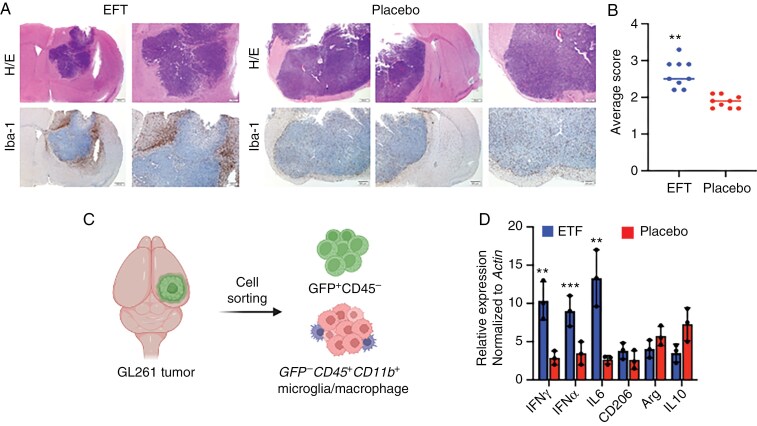
Directionally non-rotating electric field treatment (dnEFT) increased accumulation of M1-like microglia/macrophage in the tumor microenvironment. (A) Hematoxylin and Eosin (H&E) staining of dnEFT and placebo-treated GFP-labelled GL261 (GFP-GL261). C57BL/6 female mice implanted with the 4-electrode array and GFP-labeled GL261 cells were randomly assigned to either dnEFT or placebo treatment for 1 week. Subsequently, tumors were harvested for H&E (top panel) and Iba-1 staining (bottom panel). *n* = 3 for dnEFT and placebo-treated groups, representative images shown. Of note, the volume of the dnEFT-treated tumor was smaller than the placebo-treated tumor. (B) Scoring of Iba-1 staining. A total of 10 random, 400× fields were evaluated for each tumor (5 random intratumoral and 5 random peritumoral fields, see *Methods*) **P ≤ .01. (C) Schematic of microglia/macrophage isolation from dnEFT and placebo-treated GFP-GL261 tumors. (D) Expression of M1 (IFNγ, TNFα, and IL-6) and M2 (CD206, Arg, IL-10) genes in microglia/macrophage isolated from dnEFT and placebo-treated GFP-GL261 tumors. RNA was extracted from dnEFT and placebo-treated GFP-GL261 tumors and subjected to qPCR analysis for IFNγ, TNFα, IL-6, CD206, Arg, and IL10. Actin served as an internal control for the normalization and ***P ≤ .001 and **P ≤ .01.

Commercial clinical-grade percutaneous catheter-type spinal cord stimulation leads (Model No. 3186; 3 mm contact length/4 mm spacing, Abbott Neuromodulation) were purchased from Abbott Neuromodulation. A 96-well compatible 2-electrode array (Figure 2A) and 4-electrode array for murine experiments (Figure 3A) were designed by J.M. and C.C.C. and manufactured by P1 Technologies. The electric power sources (LXI DG1022Z) and connecting cables were purchased from RIGOL. For experiments using the clinical-grade electrode, an electrode was sterilely affixed inside a 10-centimeter (cm) plate and connected to the power source. Twenty-four hours after 1 × 10^6^ LN340 or H1915 cells were seeded around the affixed electrode, a directionally non-rotating electric field (dnEFT, 100 Hertz (Hz); 4 volt (V); alternating current; wave: sine) was generated between selected electrodes of the catheter until confluency was achieved in regions outside of the electric field (Figure 1B). For experiments involving the 96-well plate, openings for the electrode on the covering plate were created using a heated needle. The sterilized 2-electrode array was inserted through these openings and affixed with dual-cure resin ionomer (Cat# 031458550). Cells were seeded into the wells at 1 × 10^5^ per well and covered with media. The cover plate containing the electrodes was then placed (Figure 2B). dnEFT (100 Hz, 4 V, sine wave) was administered for 12–24 hours depending on the experiment.

### Crystal Violet Staining

The cells were washed with 1× phosphate buffered saline (PBS) and incubated in methanol (100%) for 30 minutes at room temperature (RT). Methanol was removed, and cells were then allowed to dry at RT for 10 minutes and stained with methanol containing 0.5% crystal violet solution for 30 minutes. The cells were washed multiple times in tap water, allowed to dry at RT and the images were captured using the LASX-application suit in Leica-DMi8 microscope.

### Apoptosis Assay

Cells (LN340, NHA, or MGG123) were seeded (8 × 10^4^ per well) in a 96-well plate and cultured for 24 hours. The cells were exposed to dnEFT (100 Hz, 4 V, sine wave) for 12 or 24 hours. The treated and control cells were harvested by centrifugation at 1200 rpm for 5 minutes, washed with 1× PBS, and then 1× Annexin-V binding buffer (Cat# 00-0055-43). The processed cells were resuspended in 1× Annexin-V binding buffer (100 μL) and incubated with Annexin V-FITC (Cat# 11-8005-72, Thermo Scientific) antibody (5 μL) at 37°C for 15 minutes. Cells were washed with 10 volumes of 1× Annexin-V binding buffer and resuspended in 5 μL of propidium iodide (PI) (Cat# 00-6990-42, Thermo Scientific) containing 1× Annexin-V binding buffer (200 μL). The suspensions were incubated on ice for 10 minutes and analyzed by Beckman Coulter CytoFLEX flow cytometer (C00772, Beckman Coulter). Ten thousand events were recorded for each sample, and the data analysis was performed using FlowJo software.

### Xenograft and Electrode Implantation

Animal studies were performed in accordance with the Guide for the Care and Use of Laboratory Animals (Guide for the Care and Use of Laboratory Animals, 8th edition, National Research Council (US) Committee for the Update of the Guide for the Care and Use of Laboratory Animals. Washington (DC): National Academies Press (US); 2011. ISBN-13: 978-0-309-15400-0ISBN-10: 0-309-15400-6). The animal study protocol was approved by the Institutional Animal Care and Use Committee (IACUC Protocol 2206-40091A). C57BL/6 female and male mice were used for the experiments. After induction of general anesthesia, a skin incision was made, and a burr hole was made at the tumor injection site (1.8 mm to the right of bregma) followed by durotomy. The 4-electrode array (Figure 3A) was inserted and cemented onto the murine skull. 1 × 10^4^ cells in 4 μL volume were injected through the central channel. One week after injection, tumor engraftment was verified by bioluminescence assessment (see below). The mice were then randomized to dnEFT (100 Hz, 4 V, sine wave) for 1 week or placebo (connected to the cable wire but without delivery of dnEFT). For survival studies, mice were weighed every 3 days and assessed for neurologic conditions^30^; mice reaching endpoint were sacrificed.

### Bioluminescence Imaging

Mice were anaesthetized by inhalation of 2% isoflurane and the dose was maintained throughout the imaging procedures. 150 mg/kg d-luciferin dissolved in 1× PBS was injected into the peritoneum, and the mice underwent sequential exposures in auto mode (IVIS50 imaging system) based on instructions provided by the manufacturer. The images were acquired on days 14 and 24 postinitiation of dnEFT. Bioluminescence intensity was assessed using the Living Image 3.2 software (Caliper Life Sciences). Total flux values (photons [p]/second [s]) were determined by delineating the regions of interest of uniform size on each mouse.

### Flow Cytometry

Single cell suspensions were prepared from freshly harvested green fluorescent protein (GFP) expressing GL261 tumors with and without 1 week dnEFT as previously described^29^ and stained with LIVE/DEAD Fixable Aqua Dead Cell Stain (Life Technologies), primary antibodies against CD45-Alexa700 (mouse, 30-F11) and CD11b-APC (mouse, M1/70). Sorting for ^*GFP−CD45+CD11b+*^ microglia/macrophage was performed using BD FACSAria (BD Biosciences) 11 color high-speed sorter.^29^

### Quantitative RT-PCR

Flow cytometry-sorted cells were subjected to total RNA extraction utilizing the QIAGEN RNeasy Mini Kit, followed by cDNA synthesis using 1 µg of RNA and the iScript cDNA Synthesis Kit (Bio-Rad). SYBR green-based qPCR (Bio-Rad) was performed utilizing the primers detailed in Supplementary Table 1. b-Actin served as an internal control for the normalization of mRNA levels, ensuring the accuracy of the gene expression analyses.

### Immunohistochemistry

Extracted tissues were paraffin-embedded, sectioned, mounted onto slides, and stained with hematoxylin and eosin (H&E). For Iba-1 immunohistochemistry (IHC), 4 μm sections were placed onto positively charged glass slides. The slides were deparaffinized and rehydrated. Antigen retrieval was performed. Endogenous peroxidase was blocked using 3% hydrogen peroxide followed by Dako Protein Serum Block. Primary antibody, IBA-1 (Microglia-1, Biocare cat# CP290B, 1:600 dilution) was incubated for 30 minutes at RT. Detection was achieved using Rabbit EnVision+ Kit (cat# K4003, Dako) and developed using diaminobenzidine (Dako) chromogen. Slides were counterstained with Mayer’s hematoxylin.

The IHC sections were evaluated and scored based on a previously published scoring system.^31^ In brief, for each Iba-1stained section, a total of 10 random, 400× fields were evaluated (5 random intratumoral and 5 random peritumoral fields). In each field, the density of Iba-1 positive cells was scored as 0 (no Iba-1 positive cells), 1 (Iba-1 positivity in 1%–10% of nucleated cells), 2 (Iba-1 positivity in 11%–33% of nucleated cells), 3 (Iba-1 positivity in 34%–67% of nucleated cells), or 4 (Iba-1 positivity in 67%–100% of nucleated cells).

### Statistical Analysis

Data are presented as mean ± SE. Statistical analyses were conducted using GraphPad Prism software. The statistical significance was evaluated using Student’s *t* test or 1-way ANOVA. *P* ≤ .05 was considered statistically significant.

## Results

### Antitumor Activity Associated With Directionally Non-Rotating Electric Field Treatment

We first examined the impact of dnEFT on the LN340 glioblastoma cell line in vitro. With a focus on clinical translation, a clinical-grade catheter-type electrode (Figure 1A) was aseptically affixed to a 10-cm tissue culture dish. LN340 cells were then seeded onto this dish, and an electric field (100 Hz, 4 V, alternating sine wave) was generated between electrodes 3 and 4 (Figure 1B). dnEFT was maintained throughout the 7-day experiment. We had systematically tested the biologic effects of different stimulation parameters and found that these parameters consistently reduced the viability of glioblastoma cells. Upon crystal violet staining, LN340 confluency was observed, except in the specific region corresponding to the electric field between electrodes 3 and 4. Similar results were observed when the experiment was performed using a non–small cell lung cancer H1915 cell line (Figure 1C). These findings indicate that dnEFT produced by a clinical-grade stimulator induced antineoplastic effects.

### Directionally Non-Rotating Electric Field Treatment Induces Glioblastoma Apoptosis In Vitro

A higher-throughput experimental platform is necessary to investigate the mechanism underlying dnEFT-associated antineoplastic effects. To this end, we custom-designed a 2-electrode array (Figure 2A) compatible for experimental use with 96-well tissue culture plates (Figure 2B). For these experiments, dnEFT (100 Hz, 4 V, sine wave) was administered 24 hours after LN340 cells were seeded. Cells subjected to 12 or 24 hours of dnEFT were then crystal violet stained for viability after an additional 7 days of incubation. This experiment showed that LN340 cells exposed to a dnEFT (100 Hz, 4 V, sine wave) for 12 or 24 hours exhibited compromised viability (Figure 2C). Consistent with the published literature,^32^ the temperature of the media remained unchanged throughout the duration of dnEFT delivery. Similar results were observed for U87MG and H1915 cells (Supplementary Figure 1).

To elucidate the underlying mechanism of cell death induced by dnEFT, LN340 or were harvested following exposure to dnEFT for 12 or 24 hours (100 Hz, 4 V, sine wave) and subjected to Annexin V-FITC antibody and PI staining. Subsequent flow cytometry analysis revealed a 15-fold increase in apoptosis after 12 hours of dnEFT exposure relative to the placebo-treated cells (placebo defined as exposed to the electrodes but without the delivery of dnEFT, *P* = .001). The percentage of apoptotic cells in dnEFT-treated cultures surged 25-fold compared to placebo-treated cells after 24 hours (*P* < .001, Supplementary Figure 2). We further validated these results using a patient-derived glioblastoma sphere line, MGG123.^28^ Annexin V-FITC cell counts represented in histogram demonstrated a significant increase in apoptosis in dnEFT (Figure 2D). dnEFT treatment (12 hours) of MGG123 caused a 15-fold increase in apoptosis relative to the placebo-treated cells. The percentage of apoptotic cells in dnEFT-treated cultures increased 50-fold compared to placebo-treated cells after 24 hours (*P* < .001). There was no necrosis observed by PI staining (Figure 2E). Normal human astrocytes were processed similarly and did not show apoptosis or necrosis following dnEFT treatment (Supplementary Figure 3). These findings suggest that the proapoptotic effect of dnEFT is time dependent and tumor-specific. In contrast, the proportion of necrotic cells did not change significantly after dnEFT.

### Directionally Non-Rotating Electric Field Treatment Suppressed Glioblastoma Growth In Vivo

To validate the antineoplastic effects of dnEFT in vivo, we engineered a 4-electrode array (Figure 3A and B) featuring a center channel equidistant from the electrodes. This configuration ensured that implanted tumors were centrally located within the electric field (Figure 3C). After implanting the 4-electrode array, luciferase-labeled murine glioblastoma GL261 cells were implanted through the center channel. One-week postengraftment and after confirmation of tumor engraftment by bioluminescence, the mice were randomized into dnEFT-treated and placebo-treated (ie, no dnEFT-delivered) groups for a duration of 2 weeks. Subsequently, bioluminescence assessments and physical examinations were conducted until survival endpoints.

While the bioluminescence intensities prior to dnEFT were comparable in both groups of mice, the placebo-treated cohort displayed a time-dependent increase in bioluminescence that was not observed in the dnEFT-treated mice (*n* = 8 for dnEFT- and placebo-treated groups, Figure 3D). The difference in bioluminescence was statistically significant at both the 14- and 24-day post-dnEFT time points (*P* < .001 for both) (Figure 3E). Furthermore, mice subjected to dnEFT exhibited an approximate 6-day increase in median survival compared to the placebo-treated mice (38 days vs 32.5 days, *n* = 6 for dnEFT- and placebo-treated groups, *P* < .01, Figure 3F). Importantly, these effects were consistently observed in both female (Figure 3F) and male mice (*P* < .05) (Figure 3G). These findings provide evidence supporting the in vivo efficacy of dnEFT in impeding tumor growth and improving murine survival.

### Directionally Non-Rotating Electric Field Treatment Induced Accumulation of M1-Like Microglia/Macrophage in the Tumor Microenvironment

To evaluate the impact of dnEFT on the tumor microenvironment, mice implanted with the 4-electrode array and GFP-labeled GL261 cells were randomly assigned to either dnEFT or placebo treatment for 1 week. Subsequently, tumors were harvested for IHC analysis. Hematoxylin and eosin staining of tumor sections revealed a reduction in tumor volume compared to the placebo-treated group (*n* = 3 for dnEFT and placebo-treated groups, representative images shown in Figure 4A and Supplementary Figure 4). Further, immunostaining with Iba-1 demonstrated a significant increase (*P* = .01) in microglia/macrophage accumulation within the dnEFT-treated tumors (Figure 4A and B). Isotype antibody was used as control (Supplementary Figure 5). These findings indicate not only a reduction in tumor volume but also a pronounced shift in the tumor microenvironment, marked by increased infiltration of microglia/macrophages.

M1 and M2 broadly describe the major and opposing cell states of myeloid immune cells, including microglia and macrophages.^33^ The M1 state is characterized by a proinflammatory gene signature, including the expression of IFNg, TNFa, and IL-6, and is associated with antineoplastic immune responses.^34^ In contrast, the M2 state is linked to genes (CD206, Arg, IL10) associated with immune suppression.^34^ To further characterize the nature of the myeloid infiltration, ^*GFP−CD45+CD11b+*^ microglia/macrophages (Figure 4C) were isolated from freshly resected GFP-GL261 tumors that underwent dnEFT or placebo treatment and subjected to expression profiling by RT-qPCR.^29^ In this analysis, microglia/macrophages isolated from dnEFT-treated tumors exhibited enhanced expression of M1-associated genes (IFNγ, TNFα, and IL-6) (*P* < .001) and decreased expression of M2-associated genes (CD206, Arg, IL-10) relative to those isolated from placebo-treated tumors (Figure 4D). These results suggest that dnEFT treatment triggers an accumulation of M1-like microglia/macrophages in the glioblastoma microenvironment.

## Discussion

Approximately 90% of clinical drug development endeavors fall short of achieving meaningful clinical efficacy,^35^ with each failure incurring a substantial cost of $1–2 billion and a time investment typically exceeding 10 years.^36^ Given the exorbitant expenses and high failure rate inherent in this process, there is a pressing need for alternative paradigms in therapeutic development. The foundational goal in drug development involves the design/formulation of agents that disrupt target protein, nucleic acid, or lipid function(s).^37,38^ Given the pivotal role of electrostatic interactions in nearly all facets of these functions,^1,39^ disruption of these functions through an externally imposed electric has emerged an appealing, alternative platform. Adding impetus to this is the FDA approval of directionally rotating TTF as a glioblastoma treatment. However, the modest survival benefit of TTF in most glioblastoma patients^14,16,17^ as well as the inherent limitations of the scalp electrodes^21,22^ suggest the need for further development. dnEFT through electrode(s) implanted into the tumor-bearing tissue is evaluated in this context. Rationale supporting such therapeutic paradigm include (1) the highest intensity of the EF can be generated within the tumor, thereby increasing the likelihood of therapeutic efficacy and bypassing the risks associated with scalp electrodes^19^ and (2) intracranial electrode implant is an established clinical practice for the treatment of Parkinson’s disease,^25,40^ paving a path to expedited a first-in-human translation of implanted electrode as a brain cancer treatment platform.

While the exact mechanism underlying this antitumor effect remains unclear, dnEFT ultimately triggers glioblastoma apoptosis (Figure 2), without induction of necrosis. Of note, such proapoptotic effects of dnEFT have previously been reported for other tumor types.^19^ Importantly, we validated the therapeutic impact of dnEFT in an in vivo glioblastoma model. In our murine glioblastoma model (GL261), dnEFT suppressed tumor growth (Figure 3) and induced an accumulation of microglia/macrophage with gene expression suggestive of an M1 phenotype (Figure 4). As such and in the context of the established clinical efficacy for rotating tumor treating fields, our study provides support for dnEFT delivered through implanted electrodes as a potential strategy for glioblastoma treatment.

To perform the in vivo dnEFT experiment, we had to design a novel electrode array/wire complex, as described in Figure 3A. The implanted mice can only tolerate the weight of this electrode array/wire complex for approximately a week, after which the implanted mice (without dnEFT treatment) show decreased mobility and weight loss (due to poor food intake). As such, we had to remove the wires from the mice after 1 week of dnEFT treatment and monitored the mice subsequently in the survival experiments. After detachment, wire and electrode complex could not be reconnected due to the fragility of the connection. Thus, the in vivo experiments were limited to 1 week of dnEFT treatment. Despite this limitation, we observed a significant and reproducible reduction in tumor burden (as evidenced by the decrease in bioluminescence) and improved survival in mice with glioblastoma implant. The anti-glioblastoma activity of dnEFT reported in our study is comparable to the effects reported for several other glioblastoma therapeutics in the literature.^2,41,42^ Moreover, consistent anti-glioblastoma effects were observed in both male and female mice, suggesting biological robustness and consistency.

The recruitment of microglia and macrophages exhibiting M1 antineoplastic features in response to dnEFT aligns well with emerging paradigms from recent immunotherapy cancer trials that emphasize the crucial role of anticancer immunity in achieving durable treatment responses.^5,43^ While myeloid immune cells, such as microglia and macrophages, inherently possess native, antineoplastic activities,^44^ these activities are corrupted by glioblastoma cells during carcinogenesis.^45,46^ By the time of clinically detectable tumor burden, most microglia/macrophages in the tumor microenvironment had undergone reprogramming by glioblastoma cells, adopting a protumorigenic M2 phenotype, as seen in Figure 4D and corroborated by the current literature.^47,48^ Our finding that microglia/macrophages isolated from dnEFT-treated glioblastoma remain in an M1 state is largely consistent with findings that TTF stimulate expression of TNF-α and IL-6 in RAW 264.7 murine macrophage cells.^49^ Others have also reported that dnEF induce myeloid expression of M1-associated genes.^5^ These results offer promise for dnEFT-associated antitumor immunity and introduce the possibility of synergistic effects between dnEFT and select immunotherapeutic agents.^50,51^

Recent advances in the field of cancer neuroscience have revealed insights into the potential role of neuron–tumor interactions in the pathogenesis of glioblastoma.^52^ Oncogenic and tumor suppressor mutations in glioblastoma are associated with remodeling of synaptic constituencies in the glioblastoma tumor microenvironment.^53^ Optogenetic induction of neuronal firing is associated with release of growth factors that stimulate glioblastoma growth in preclinical models.^54^ Our work adds to this literature by demonstrating that direct exposure of glioblastoma to specified electric fields induced glioblastoma apoptosis and accumulation of antitumoral microglia in the tumor microenvironment. Whether the effects observed in this study are pertinent to the natural electric fields that arise during normal biologic processes^55^ remains an open question.

As this study is designed as a proof-of-principle for the therapeutic effect of dnEFT, it cannot definitively address the merits of dnEFT relative to TTF. Further, while the utilization of currently available clinical-grade electrodes may facilitate translation of a proof-of-principle human study, it is crucial to acknowledge that the currently available electrodes have not been optimized as a cancer treatment modality. For instance, the existing clinical electrodes generate an electric field of less than 1 cm in maximal width, a dimension insufficient to address tumor burdens typically encountered in the clinical setting. Improved electrode design can additionally facilitate the targeted delivery of directionally rotating TTF as well as dnEFT. Furthermore, identification of the optimal stimulation parameters for an antitumor dnEFT remains an unresolved issue. The available literature,^5–7,9,10,18,56,57^ as well as our pilot data, suggests that distinct biological processes are impacted by electric fields defined by differing parameters (eg, frequency, wave form, and so on). Consequently, the alignment of tumor susceptibility to the specific parameters of the dnEFT delivered may impact clinical efficacy.^58^ A corollary to this hypothesis is that a single electrode may function as a “multi-drug” (https://patents.google.com/patent/US11623085B2/en?oq=US11623085B2) based on the stimulation parameters applied. Induction of electric fields may also augment tumor uptake of nucleic acid-based therapeutics through “electroporation”^5^ as a form of gene therapy. These intriguing possibilities await exploration in future investigations.

## Supplementary Material

vdae121_suppl_Supplementary_Material
