# Correction to: Directionally non-rotating electric field therapy delivered through implanted electrodes as a glioblastoma treatment platform: A proof-of-principle study

**DOI:** 10.1093/noajnl/vdaf125

**Published:** 2025-06-28

This is a correction to: Jun Ma, Shilpi Singh, Ming Li, Davis Seelig, Gregory F Molnar, Eric T Wong, Sanjay Dhawan, Stefan Kim, Logan Helland, David Chen, Nikos Tapinos, Sean Lawler, Gatikrushna Singh, Clark C Chen, Directionally non-rotating electric field therapy delivered through implanted electrodes as a glioblastoma treatment platform: A proof-of-principle study, *Neuro-Oncology Advances*, Volume 6, Issue 1, January-December 2024, https://doi.org/10.1093/noajnl/vdae121

The following changes have been made to the originally published manuscript. The tenth co-author’s name is emended to read: “Hsien-Chung Chen” instead of “David Chen”. Their affiliation is also emended to read: “Department of Neurosurgery, Shuang Ho Hospital, Taipei Medical University, Taipei, Taiwan” instead of: “Department of Neurosurgery, Taiwan Medical University, Taipei, Taiwan”.

