# Supplementary material for: Directionally non-rotating electric field therapy delivered through implanted electrodes as a glioblastoma treatment platform: A proof-of-principle study
Source: Neurooncol Adv. 2024 Jul 13;6(1):vdae121. doi: 10.1093/noajnl/vdae121 (PMC11327618; doi:10.1093/noajnl/vdae121)
Supplement: vdae121_suppl_Supplementary_Material [file vdae121_suppl_supplementary_material.docx]

**Directionally non-rotating electric field therapy (dnEFT) delivered through implanted electrodes as a glioblastoma treatment platform: a proof-of-principle study**

**Authors:** Jun Ma, MD^1^; Shilpi Singh, PhD^1^; Ming Li, PhD^1^; Davis Seelig, DVM, PhD^2^; Gregory F Molnar, PhD^3^; Eric T Wong, MD^4^, Sanjay Dhawan, MD^1^, Stefan Kim, MD^1^; Logan Helland, MD^1^; David Chen MD^5^, Nikos Tapinos, MD PhD^6^, Sean Lawler, PhD^7^, Gatikrushna Singh, PhD^1^*; Clark C. Chen, MD, PhD^1,6^*

**Supplementary Table 1: Primers utilized for quantitative RT-PCR.**

| **Target gene (mouse)** | **Forward primer** | **Reverse primer** |
| --- | --- | --- |
| IFNγ | 5’ CAGCAACAGCAAGGCGAAAAAGG 3’ | 5’ TTTCCGCTTCCTGAGGCTGGAT 3’ |
| TNFα | 5’ CAGAAACACAAGATGCTGG 3’ | 5’ CAAAAGAGGAGGCAACAAGG 3’ |
| IL6 | 5’ TCTGCAAGAGACTTCCATCC 3’ | 5’ TTAGCCACTCCTTCTGTGAC 3’ |
| CD206 | 5’ GTTCAGCTATTGGACGCGAG 3’ | 5’ GAATCTGACACCCAGCGGAA 3’ |
| ARG1 | 5’AACACGGCAGTGGCTTTAACC 3’ | 5’ GGTTTTCATCTGGCGCATTC 3’ |
| IL10 | 5’ CCTTAATGCAGGACTTTAAGGGTTA 3’ | 5’ TTACACTCGCCCCCTTTGC 3’ |
| b-Actin | 5’ CATTGCTGACAGGATGCAGAAGG 3’ | 5’ TGCTGGAAGGTGGACAGTGAGG 3’ |

**Supplementary Figure 1: Directionally non-rotating electric field treatment (dnEFT) induces cell lysis.**

U87MG and H1915 cells were exposed to a dnEFT (100 Hz, 4 V, sine wave) for 24 h. Photomicrographs were taken through the Leica DMi8 inverted microscope.


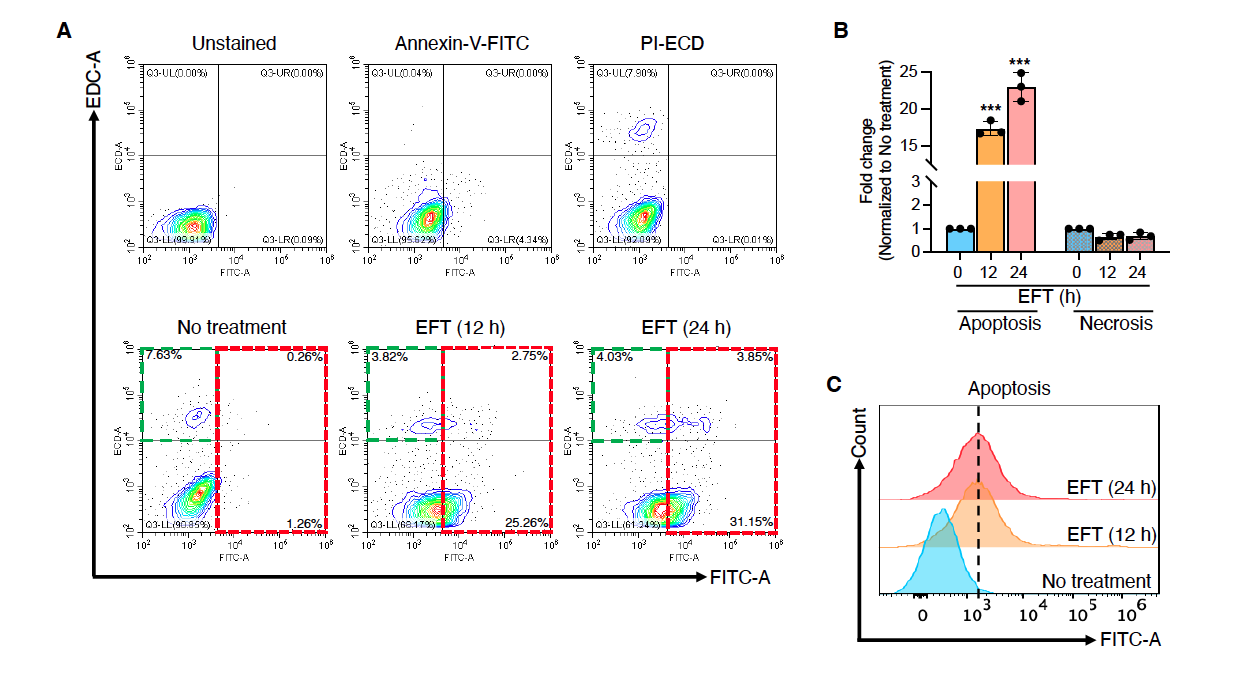


**Supplementary Figure 2: Directionally non-rotating electric field treatment (dnEFT) induces glioblastoma apoptosis in LN340 cells.**

**(A)** Flow-cytometric analysis of Annexin V-FITC and propidium-iodide stained LN340 cells with or without dnEFT (100 Hz, 4 V, sine wave, 12 or 24 h). The gate was determined by the unstained, single stained (Annexin V-FITC and PI- ECD) cell populations. Lower right (LR) and upper right (UR) quadrants (red line) showed Annexin V-FITC stained early and late apoptotic and upper left (UL) quadrants (green dotted line) shows propidium iodide-stained necrotic cells. **(B)** The fold change of apoptotic and necrotic cells population was determined by the normalization with unstimulated cell population. The bar graph represents the mean standard deviation of three independent biological replicates. Statistical significance: *** p≤0.001. **(C)** Histogram represents the apoptotic cell distribution of no-treatment, EFT treatment of 12 h and 24 h.


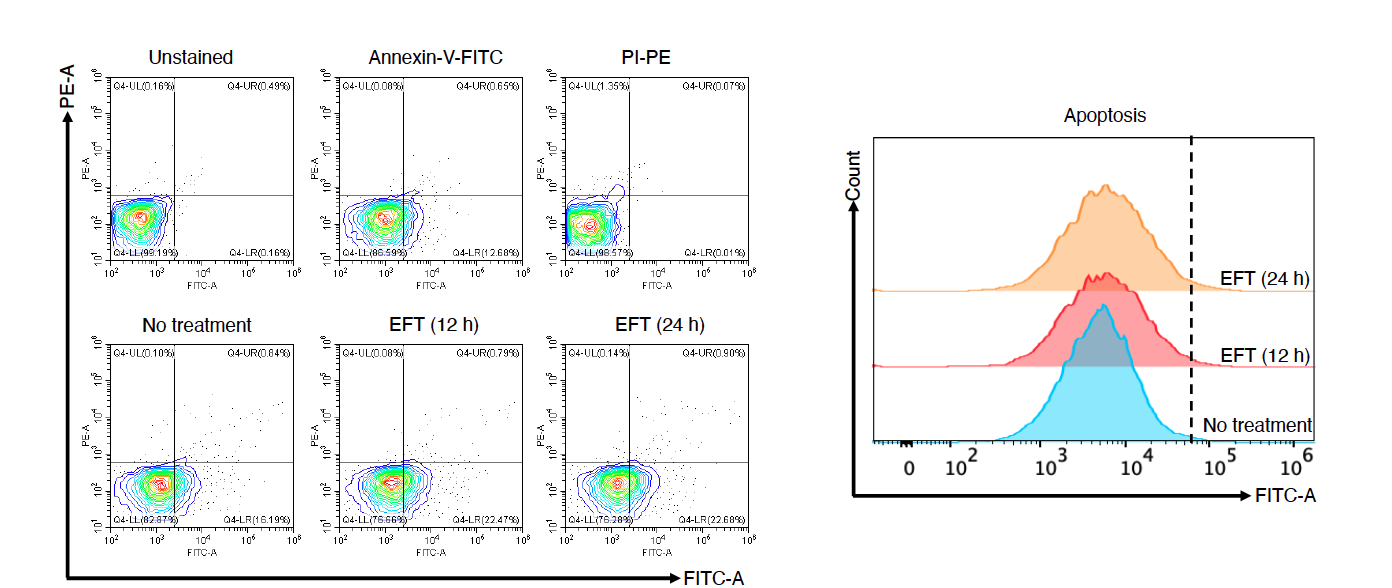


**Supplementary Figure 3: Directionally non-rotating electric field treatment (dnEFT) did not affect normal human astrocytes.**

Flow-cytometric analysis of Annexin V-FITC and propidium iodide-stained normal human astrocytes (NHA) cells with or without dnEFT (100 Hz, 4 V, sine wave, 12 or 24 h). The gate was determined by the unstained, single stained (Annexin V-FITC and PI- PE) cell populations. Lower right (LR) and upper right (UR) quadrants (red line) showed Annexin V-FITC stained early and late apoptotic and upper left (UL) quadrants (green dotted line) shows propidium iodide-stained necrotic cells. Histogram represents the apoptotic cell distribution of no-treatment, EFT treatment of 12 h and 24 h.

**Supplementary Figure 4: Directionally non-rotating electric field treatment (dnEFT) reduced tumor volume.**

Representative hematoxylin and Eosin (H&E) staining of dnEFT and placebo treated GFP-labelled GL261 (GFP-GL261). C57BL/6 female mice implanted with the four-electrode array and GFP-labeled GL261 cells were randomly assigned to either dnEFT or placebo treatment for one week. Subsequently, tumors were harvested for H&E (top panel) and Iba-1 staining (bottom panel). H&E shows the volume of the dnEFT treated tumor was smaller than the placebo treated tumor. Immunostaining with Iba-1 demonstrated a significant increase (p=0.01) in microglia/macrophage accumulation within the dnEFT-treated tumors. Of note, the tumors shown here are distinct from those shown in **Figure 4A**.

**Supplementary Figure 5: Isotype antibody staining.**

Representative images from control isotype antibody staining for the experiments described in **Figure 4A**. Staining of sections from dnEFT and placebo treated tumor samples are shown.
